# Protection against a chlamydial respiratory challenge by a chimeric vaccine formulated with the *Chlamydia muridarum* major outer membrane protein variable domains using the *Neisseria lactamica* porin B as a scaffold

**DOI:** 10.1038/s41541-020-0182-9

**Published:** 2020-05-08

**Authors:** Delia F. Tifrea, Sukumar Pal, Jeff Fairman, Paola Massari, Luis M. de la Maza

**Affiliations:** 1 Department of Pathology and Laboratory Medicine, University of California, Irvine, Medical Sciences I, Room D440, Irvine, California 92697-4800 USA; 2 Sutrovax, Inc., 400 E Jamie Court, Suite 205, South San Francisco, California 94080 USA; 3 Department of Immunology, Tufts University School of Medicine, Jaharis, 512C 150 Harrison Avenue, Boston, Massachusetts 02111 USA

**Keywords:** Protein vaccines, Protein vaccines

## Abstract

*Chlamydia trachomatis* is the most frequently detected sexually transmitted bacterial pathogen in the world. Attempts to control these infections with screening programs and antibiotics have failed and, therefore, a vaccine is the best approach to control this epidemic. The *Chlamydia* major outer membrane protein (MOMP) is the most protective subunit vaccine so far tested. Protection induced by MOMP is, in part, dependent on its tertiary structure. We have previously described new recombinant antigens composed of the *Neisseria lactamica* PorB engineered to express the variable domains (VD) from *Chlamydia muridarum* MOMP. Here we tested antigens containing each individual MOMP VD and different VD combinations. Following immunization, mice were challenged intranasally with *C. muridarum*. Our results show that three constructs, PorB/VD1–3, PorB/VD1–4, and PorB/VD1–2–4, elicited high serum IgG titers in vivo, significant IFN-γ levels upon T cells re-stimulation in vitro, and evidence of protective immunity in vivo. PorB/VD1–3, PorB/VD1–4, and PorB/VD1–2–4 immunized mice lost less body weight, had lighter lungs, and decreased numbers of inclusion forming units (IFUs) in lungs than other PorB/VD construct tested and mock PBS-immunized mice. These results suggest that this approach may be a promising alternative to the use of MOMP in a *Chlamydia* vaccine.

## Introduction

*Chlamydia trachomatis* is the most frequently isolated sexually transmitted bacterial pathogen worldwide^1,2^. In addition, it also produces respiratory, gastrointestinal and ocular infections with a wide range of clinical presentations^3–7^. In women, most of the genital infections are asymptomatic^5,7^. However, in some patients, acute cervicitis and urethritis, and long-term sequelae including pelvic inflammatory disease, chronic abdominal pain, ectopic pregnancy, and infertility occur^8–13^. Public health efforts to control genital infections by screening individuals at risk and implementing antibiotic therapy have not yielded the expected results and the number of genital chlamydial infections continues to increase^14,15^. Therefore, a vaccine is likely the most effective approach to control this pathogen^16–24^.

In countries with poor sanitary conditions, ocular *C. trachomatis* infections can lead to scarring of the cornea and blindness (trachoma)^6^. When *C. trachomatis* was identified as the cause of trachoma, vaccine trials were conducted in humans and non-human primates using live or inactivated organisms^3,6,25^. Several conclusions were reached from those trials. Some vaccines elicited serovar/serogroup specific but short-lived protection (1–3 years). In addition, a few vaccinated individuals had increased number of infections or developed a hypersensitivity reaction upon reexposure to the pathogen^25–29^. Although the mechanisms underlying these negative effects are not understood, the possibility that one of the bacterial components present in the whole organism was inducing an autoimmune reaction was considered^3,6,30^. For these reasons, the search for a subunit vaccine was initiated.

*C. trachomatis* includes 15 major serovars (A-L3) divided into a B- (B, Ba, E, D, L1, and L2) and a C-immunocomplex (C, J, H, I, and A), and two minor related complexes (K and L3) and (G, F)^31–33^. Serovars A-C cause ocular infections, (D-K) produce oculo-genital infections, and the L serovars are the etiological agents of lymphogranuloma venereum. DNA sequencing of the chlamydial genome and phylogenetic analysis suggested that the *C. trachomatis* major outer membrane protein (MOMP) was the antigen responsible for the serovar/serogroup protection observed during the trachoma vaccine trials^34,35^.

MOMP accounts for 60% of the elementary body (EB) outer membrane mass, has a porin function, and it is predicted to have a native trimeric structure^36,37^. A topology model of native MOMP (nMOMP) has suggested a β-barrel core with eight surface-exposed loops and eight short cytoplasmic loops, consistent with the structure of other bacterial porins^37,38^. Areas of high sequence variability (variable domains, VDs) have been identified within four of the eight surface-exposed loops, flanked by regions of constant amino acid sequences (CDs)^38^. The amino acid sequence of the VD defines each serovar. MOMP is highly antigenic^39^ and has been shown to elicit robust protection against genital and respiratory challenge in mice and against ocular challenges in non-human primates^40–43^. Immunization with purified nMOMP led to decreased vaginal shedding and inflammatory responses in the upper genital tract in a mouse *Chlamydia* vaginal challenge model^44–46^. Other chlamydial proteins that have been explored as vaccine antigens in animal models of genital and respiratory challenge with *Chlamydia muridarum* include the *Chlamydia* protease-like activity factor (CPAF), the polymorphic membrane proteins (Pmps), and the plasmid glycoprotein 3 (Pgp3)^17,23,24,42^. However, only MOMP elicited protection against long-term sequelae, specifically infertility^17,23,24,40,42,47–49^. The protective effect of nMOMP is attributed to induction of both neutralizing antibodies and CD4+ T-cell-mediated production of interferon (IFN)-γ^43,50^. Protective B-cell epitopes have been mapped to the VDs within loops 2, 3, 5, and 6 of both *C. trachomatis* and *C. muridarum* MOMP, and predicted and known T-cell epitopes are located within the adjacent CD regions, some of which also span through the VDs^51–54^.

Attempts to refold rMOMP into its trimeric native conformation have so far failed. Madico et al.^55^ engineered novel recombinant chimeric antigens composed of the *Neisseria lactamica* PorB porin in which entire loops were replaced by *C. muridarum* MOMP loops containing the immunogenic VD regions. This strategy takes advantage of the similarities between the structures of *N. lactamica* PorB and *C. muridarum* MOMP, allowing to obtain chimeric proteins in which the VDs are expressed and presented for immune recognition in a trimeric-structured folded scaffold^55^. Here, the PorB/VD chimeric antigens were examined for their ability to induce protective immunity against *Chlamydia* infection. Our results demonstrated that some of the PorB/VD constructs elicited humoral and cell-mediated MOMP-specific immune responses that protected mice against a respiratory challenge with *C. muridarum*.

## Methods

### Stocks of *C. muridarum*

The *C. muridarum* (strain NiggII; previously called *C. trachomatis* mouse pneumonitis biovar) was purchased from the American Type Culture Collection and grown in HeLa-229 cells^36^. *C. muridarum* EBs were purified and stored at −80 °C in sucrose phosphate-glutamate buffer^36^.

### Purification of *C. muridarum* recombinant MOMP

The gene of the *C. muridarum* MOMP (GenBank, accession number AE002272, X63409), without the leading sequence, was amplified by the PCR and cloned into the pET-45b vector (Novagen, Madison, WI). For expression, *Escherichia coli* BL21 (DE3) was transformed with the plasmid containing the *C. muridarum* MOMP sequence. The recombinant protein was extracted from the *E. coli* inclusion bodies as described by Marston^56^. The pellet of MOMP was solubilized in TEN buffer with 8 M urea, 0.1 mM phenylmethylsulfonyl fluoride (PMSF), and 0.02 mM dithiothreitol (DTT) to a concentration of 10 mg/ml. Following solubilization, MOMP was loaded onto a Sephacryl-S-300 column (1 × 50 cm; Sigma), which was pre-equilibrated with 100 mM Tris-HCl, pH 8.0, 200 mM NaCl, 10 mM EDTA, 0.2 mM DTT, and 0.05% Zwittergent 3–14 (Anatrace, Inc., Maumee, OH), and the peak fractions were pooled^41^. Before immunization, MOMP was dialyzed against phosphate-buffered saline (PBS) (pH 7.4) with 0.05% Zwittergent 3–14 (Anatrace, Inc.).

### Purification of recombinant PorB/VD antigens

Cloning of the recombinant PorB/VD antigens in *E. coli* using a pET17b plasmid, protein expression, and purification were previously described^55^ using plasmids produced by GenScript based on the sequence of each gene construct. Briefly, after transforming *E. coli* BL21 (DE3) and screening on Luria Bertani (LB) plates with 50 µg/ml carbenicillin, colonies were expanded in liquid LB with carbenicillin (100 µg/ml). The presence of *porB/VD* genes was confirmed by DNA digestion with NdeI and BamHI. Positive colonies were grown overnight at 37 °C and induced with isopropyl β-d-1-thiogalactopyranoside (0.2 mM final concentration) for 3–4 h for protein expression. PorB/VD proteins were recovered in inclusion bodies. Bacteria were lysed in TEN buffer (50 mM Tris-HCl, 1 mM EDTA, 100 mM NaCl pH 8.0) containing lysozyme, deoxycholate, and PMSF, followed by DNase I treatment and sonication. Suspensions were centrifuged, pellets were resuspended in 5 ml of TEN buffer with 8 M urea for further sonication followed by addition of 5 ml of 10% Zwittergent 3–14 (Anatrace, Inc.). The protein suspension was separated by ion exchange column chromatography on a DEAE Sepharose CL-6B column and a CM-Sepharose column in tandem pre-equilibrated in 50 mM Tris, 10 mM EDTA, 0.05% Zwittergent 3–14 (Anatrace, Inc.), and 0.02% azide (pH 8.0). The flow through was collected, precipitated with ethanol (80% v/v, final concentration), and resuspended in 100 mM Tris, 10 mM EDTA, 0.2 M NaCl, 5% Zwittergent 3–14 (Anatrace, Inc.), 0.02% azide (pH 8.0) for subsequent loading on a Sephacryl-S-300 gel filtration column pre-equilibrated with 100 mM Tris, 10 mM EDTA, 0.2 M NaCl, 0.05% Zwittergent 3–14 (Anatrace, Inc.), and 0.02% azide (pH 8.0). The porin-containing fractions were identified by Coomassie stain of SDS-polyacrylamide gel electrophoresis, collected, and precipitated with ethanol as above, followed by resuspension in 50 mM Tris, 10 mM EDTA, 5% Zwittergent 3–14 (Anatrace, Inc.), and 0.02% azide (pH 7.5). The protein suspension was loaded on a Matrex Cellufine Sulfate column pre-equilibrated with 50 mM Tris, 10 mM EDTA, 0.05% Zwittergent 3–14, and 0.02% azide (pH 7.5). The column was washed with the same buffer and proteins were eluted with a 0.2–0.5 M NaCl linear gradient. The porin-containing fractions were collected, precipitated as above, and resuspended in 10% D-octyl-glucoside (Anatrace, Inc. Maumee, OH) in 10 mM HEPES pH 7.2, followed by extensive dialysis against PBS/0.02% sodium azide for proteosome formation. All the purification and dialysis steps were performed at room temperature and the purified proteins were stored at 4 °C. Protein concentration was measured by BCA protein assay (Pierce) as per the manufacturer ‘s protocol. The panel of antigens included constructs that expressed individual MOMP loops containing each VD, as well as two (e.g., PorB/VD1–3), three (e.g., PorB/VD1–2-4), or all four (PorB/4 VDs) loops simultaneously, to obtain constructs expressing multiple VDs (Supplementary Fig. 1)^57–61^. Purified recombinant *N. lactamica* PorB was used as a negative control in the pilot experiment.

### Mice immunization

Three-week-old female BALB/c (H-2^d^) mice were purchased from Charles River Laboratories. The mice were maintained at the University of California, Irvine (UCI) Vivarium in accordance with the NIH and UCI guidelines. Each mouse was vaccinated, by both the intramuscular and subcutaneous routes, with a total of 10 µg of purified PorB/VD antigens, three times at 2-week intervals. CpG-1826 (10 µg/mouse/immunization; 5′-TCCATGACGTTCCTGACGTT-3′; Trilink Biotechnologies, Inc., San Diego, CA) and Montanide ISA 720 VG (Seppic, Inc, Fairfield, NJ; at a 30:70 volume ratio of MOMP plus CpG to Montanide) were used as adjuvants^41,48,62^. Montanide was delivered only systemically. Positive controls received *C. muridarum* rMOMP with the same adjuvants and negative controls included PBS and *N. lactamica* PorB plus the adjuvants. *S*era were collected before the first immunization and the day before each immunization and were stored at −20 °C until use. Initially, five mice per group were immunized to test all the constructs and antigens showing a protective effect in a pilot challenge study (based on body weight, lungs weight, and number of *C. muridarum* inclusion forming unit (IFU) recovered from the lungs) were tested again in a larger mice group to confirm the results (Table 1). Each experimental group included 10–12 mice.

**Table 1:** Disease burden and yields of *C. muridarum* IFU in the lungs at D10 p.c.

Immunization group	% Body weight change (mean ± 1 SE)	Lungs weight (g) (mean ± 1 SE)	Median number IFU recovered from lungs (min–max) × 10^6^
PorB/VD1	−25.17 ± 2.66	0.31 ± 0.03	1382 (118–10,042)
PorB/VD3	−21.78 ± 2.81	0.33 ± 0.01	777 (68–1457)
PorB/VD4	−23.99 ± 2.09	0.28 ± 0.01	4530 (102–6191)
PorB/VD1–2	−27.91 ± 2.88	0.31 ± 0.03	453 (287–1163)
PorB/VD1–3	−13.99 ± 1.52^a^	0.26 ± 0.01^a^	59 (4–672)^b^
PorB/VD1–4	−17.35 ± 2.41^a^	0.28 ± 0.01	66 (5–1374)^b^
PorB/VD1–3 + PorB/VD1–4	−18.68 ± 3.37	0.28 ± 0.01	681 (6–9891)
PorB/VD2–3	−27.67 ± 2.77	0.31 ± 0.02	5059 (4455–16,384)
PorB/VD2–4	−23.01 ± 1.44	0.31 ± 0.01	3700 (2341–5738)
PorB/VD3–4	−22.82 ± 1.56	0.30 ± 0.01	6720 (861–8834)
PorB/VD1–2–3	−22.54 ± 4.90	0.30 ± 0.02	317 (8–876)
PorB/VD1–2–4	−13.20 ± 2.86^a^	0.27 ± 0.01	113 (43–1027)^b^
PorB/VD1–3–4	−19.46 ± 4.90	0.26 ± 0.01	378 (8–906)
PorB/VD2–3–4	−26.04 ± 1.27	0.28 ± 0.01	4832 (906–13,590)
PorB/4 VDs	−21.66 ± 5.05	0.29 ± 0.02	551 (5–4983)
NL PorB	−18.70 ± 1.55	0.30 ± 0.01	264 (181–559)
rMOMP/Z3–14	−6.49 ± 1.36	0.21 ± 0.01	1 (0.02–228)
PBS	−23.37 ± 1.31	0.29 ± 0.01	2190 (158–9136)

^a^*P* < 0.05 by the Student’s *t*-test compared with the PBS group.

^b^*P* < 0.05 by the Mann–Whitney test compared with the PBS group.

### Ethics statement

The mice were maintained at the UCI Vivarium in accordance with the NIH and UCI guidelines. Killing was performed following the recommendations of the Panel of Euthanasia of the American Veterinary Medical Association. All procedures, methods, and the experimental plan were approved by the UCI Institutional Animal Care and Use Committee.

### ELISA antibody titers

Prior to the respiratory challenge, sera from immunized mice were used to quantify the levels of antibodies by enzyme-linked immunosorbent assay (ELISA)^40,63^. Multiwell plates were coated with *C. muridarum* EB (1 μg/well) or rMOMP (0.1 μg/well) and incubated with serial dilutions of preimmune and immune sera, followed by incubation with horseradish peroxidase-conjugated goat anti-mouse IgG (KPL, catalog number 474–1806 diluted 1:4000), and IgG1 and IgG2a (BD Pharmingen, catalog numbers 559626 and 553391, respectively, diluted 1:1000) antibodies. Chromogenic substrate detection at OD_405_ was performed using an EIA reader (Labsystem Multiscan, Helsinki, Finland) and geometric mean titers (GMTs) were expressed as the reciprocal of the dilution^40,63^.

### Chlamydia in vitro neutralization

In vitro neutralization assays were performed as follows^64^. *C. muridarum* (1 × 10^4^ IFUs) was incubated for 1 h with preimmune and immune mouse sera, diluted serially with Ca^2+^ and Mg^2+^-free PBS pH 7.2 supplemented with 5% guinea pig serum as a source of complement. Following incubation for 45 min at 37 °C, the mixtures were inoculated by centrifugation into HeLa-229 cells grown on shell vials. After 30 h at 37 °C, the monolayers were fixed and stained with a pool of monoclonal antibodies^63^. The titer of a sample is the dilution that yielded 50% neutralization relative to the control serum from pre-immunized mice.

### Mapping of linear B-cell epitopes

Specific antibodies to MOMP linear epitopes in sera from immunized mice were also measured by ELISA^43^. Overlapping 25-mers, corresponding to the mature *C. muridarum* MOMP amino acid sequence (with peptide 25 overlapping the N and C terminus of MOMP), were synthesized (SynBioSci., Livermore, CA)^65,66^. The peptides were adsorbed onto high-binding affinity ELISA plates (1 µg/well) in triplicates and serum IgG antibody levels were determined^67^.

### *C. muridarum*-specific cellular immune responses following vaccination

Cellular immune responses were measured using splenocytes collected from vaccinated mice the day prior to challenge^63^. Splenic T cells, purified using nylon wool (>95% purity), were stimulated with *C. muridarum* EB in the presence of antigen-presenting cells (APCs) pre-prepared by irradiation (3300 rads, ^137^Cs) of syngeneic splenocytes. APCs (1.25 × 10^5^ cells) were incubated with EB at 1:1 ratio for 2 h at 37 °C in round-bottom 96-well plates (Costar, Corning, Inc.), followed by addition of T cells also at a ratio of 1:1. Concanavalin A (5 μg/ml) served as a positive stimulant and cell culture medium (RPMI with 10% fetal bovine serum) used as a negative control. Levels of IFN-γ and interleukin (IL)-6 in the culture supernatants were determined using commercial kits (BD Pharmingen, San Diego, CA)^68^.

### Intranasal *C. muridarum* mice challenge and evaluation of infection and disease

Four weeks after the last immunization, mice were challenged intranasally (i.n.) with 10^4^ IFU of *C. muridarum*^41,69^. The mice weight was recorded during 10 days post challenge (d.p.c.), after which the animals were killed and the lungs were collected. The weight of the lungs was recorded prior to tissue homogenization and serial tenfold dilutions of homogenized tissues were used to inoculate Hela-229 cell monolayers^41,70^. Cell cultures were incubated for 30 h in a 5% CO_2_ 37 °C incubator, stained with *C. muridarum*-specific monoclonal antibodies, and IFUs were counted using a light microscope^63^. The limit of detection was <50 *C. muridarum* IFUs/lungs/mouse.

Following homogenization of the lungs, the tissues were centrifuged for 10 min at 1000 × *g* and the supernatants collected for quantification of IFN-γ and *C. muridarum*-specific IgA. The titers of *C. muridarum*-specific IgA and levels of IFN-γ in lung’s supernatants at D10 post challenge (p.c.) were determined by an ELISA as described^71^.

### Statistical analyses

The Mann–Whitney’s *U*-test was used to compare the number of *C. muridarum* IFU and the Student’s *t*-test (two-sided) was performed to compare humoral and cellular responses, lungs weight, and body weight changes of mice. Repeated-measures analysis of variance (ANOVA) analyses were conducted to compare changes in mean body weight over the 10 days of observation following the intranasal challenge.

## Results

### Screening of PorB/VDs vaccine constructs

To ascertain the protective ability of the recombinant PorB/VD constructs, an initial in vivo screening was performed using groups of five mice each (Table 1). Animals were immunized with constructs expressing individual MOMP loops containing each VD (i.e., PorB/VD1, PorB/VD2, etc.), combination of two loops (i.e., PorB/VD1–2, etc.), three loops (i.e., PorB/VD1–2–3, etc.), or all four loops simultaneously (PorB/4 VDs). Two negative controls groups, one immunized with PBS plus the adjuvants and another one with *N. lactamica* PorB with the adjuvants were included in the study. The positive control group was immunized with *C. muridarum* rMOMP.

Four weeks after completion of the immunization schedule, the mice were challenged i.n. with *C. muridarum* to assess protection from infection by measuring body weight and lung weight loss at D10 p.c. and number of *C. muridarum* IFUs recovered from the lungs. Based on these parameters, our pilot study determined that three PorB/VD constructs had a protective effect PorB/VD1–3, PorB/VD1–4, and PorB/VD1–2–4. Thus, these constructs and a combination of PorB/VD1–3 + PorB/VD1–4 were selected for additional testing in mice.

### Characterization of humoral responses induced by vaccination with PorB/VD1–3, PorB/VD1–4, and PorB/VD1–2–4

The antibody titers to whole *C. muridarum* EBs and purified rMOMP elicited by vaccination with the PorB/VD constructs were measured by an ELISA in mouse sera collected the day before the intranasal challenge. Sera from PBS-immunized mice was used as background control. The highest ELISA IgG antibody GMTs to *C. muridarum* EB were observed in mice vaccinated with PorB/VD1–2-4 (3733) and the lowest titer in response to PorB/VD1–4 immunization (1728) (Table 2). High IgG GMTs to EBs were determined in sera from rMOMP-immunized mice (102,400) and no *C. muridarum* EB-specific IgGs were detected in the sham-immunized mice group (PBS). To assess whether the immune responses were Th1 or Th2 biased, the ratio of IgG2a/IgG1 was calculated. As shown in Table 2, except for PorB/VD1–2–4, the PorB/VD antigens elicited Th2-biased immune responses when using EB as the antigen. Mice vaccinated with rMOMP developed Th1-biased responses.

**Table 2:** Vaccine induced antibody responses in sera the day before the *C. muridarum* intranasal challenge.

Immunization groups	Anti EB serum GMT (range)				Neutralizing titer GMT (range)	Anti-MOMP serum GMT (range)			
	IgG	IgG1	IgG2a	IgG2a/IgG1		IgG	IgG1	IgG2a	IgG2a/IgG1
PorB/VD1–3	2963^a^ (1600–6400)	1600^a^ (100–6400)	283^a^ (100–800)	0.2	50 (<50–100)	129,016^a^ (102,400–204,800)	81,275^a^ (51,200–102,400)	11,404^a^ (6400–25,600)	0.1
PorB/VD1–4	1728^a^ (400–6400)	741^a^ (100–6400)	606^a^ (100–3200)	0.8	<50 (<50–<50)	64,508^a^ (12,800–204,800	36,204^a^ (6400–204,800)	7184^a^ (800–25,600)	0.2
PorB/VD1–3 + PorB/VD1–4	1600^a^ (800–3200)	2177^a^ (400–6400)	159^a^ (100–400)	0.1	<50 (<50–50)	129,016^a^ (102,400–204,800)	91,228^a^ (51,200–204,800)	11,404^a^ 3200–51,200)	0.1
PorB/VD1–2–4	3733^a^ (3200–6400)	317^a^ (100–800)	1600^a^ (200–6400)	5.0	533 (200–800)^a^	204,800^a^ (204,800–204,800)	36,204^a^ (25,600–51,200)	102,400^a^ (51,200–204,800)	2.8
rMOMP/Z3–14	102,400 (102,400–102,400)	5080 (1600–12,800)	40,637 (25,600–51,200)	8.0	1600 (800–3200)	409,600 (204,800–819,200)	102,400 (51,200–409,600)	516,064 (409,600–819,200)	5
PBS	<100	<100	<100		<50	<100	<100	<100	

*GMT* geometric mean titer.

^a^*P* < 0.05 by the Student’s *t*-test compared with the PBS group.

Sera antibody titers induced by immunization with PorB/VDs against rMOMP were much higher than those against EBs (Table 2) and retained a similar Th2-biased IgG2a/IgG1 ratio. As expected, sera from mice vaccinated with rMOMP had very high antibody GMT (409,600) to rMOMP and a Th1-biased response, whereas mice inoculated with PBS did not develop antibodies against rMOMP.

To assess the ability of anti-PorB/VD sera to neutralize *C. muridarum*, an in vitro neutralization assay was used. As shown in Table 2, immunization with PorB/VD1–2-4 induced neutralizing antibodies (GMT: 533), whereas the other constructs did not. rMOMP vaccination elicited a GMT of 1600.

**Fig. 1:**
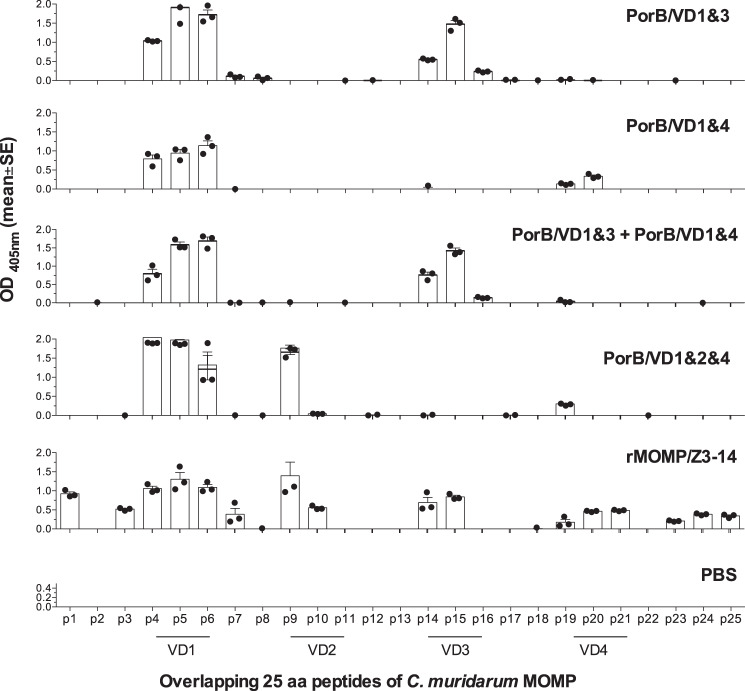
Binding of serum antibodies to synthetic *C. muridarum* MOMP peptides. Serum samples from immunized mice were collected the day before the i.n. challenge and their reactivity to 25-mer peptides corresponding to the *C. muridarum* mature MOMP were analyzed by ELISA.

To determine whether vaccination with the PorB/VD constructs elicited antibodies to specific B-cell epitopes, overlapping *C. muridarum* MOMP peptides were used as antigens (Fig. 1). Sera from mice immunized with the PorB/VD1–3 construct recognized the VD1 and VD3 of MOMP, and sera from mice immunized with the PorB/VD1–4 construct recognized VD1 and VD4. Interestingly, sera from mice immunized with PorB/VD1–3 + PorB/VD1–4 bound VD1 and VD3 but not VD4 peptides. Immunization with PorB/VD1–2-4 elicited antibodies to the three VDs. Sera from mice vaccinated with rMOMP recognized all four VD, while PBS-immunized mice failed to bind any peptide.

### Characterization of cellular responses induced by vaccination with PorB/VD1–3, PorB/VD1–4, and PorB/VD1–2–4

To evaluate induction of cellular immunity, splenic T cells were collected from a subset of mice 1 day prior to the intranasal challenge (4 weeks after the last immunization) and stimulated in vitro with *C. muridarum* EB or rMOMP. Cytokine levels were measured by ELISA of the culture supernatants and, as shown in Table 3, the highest levels of IFN-γ were observed in T-cell supernatants from mice vaccinated with PorB/VD1–3 and stimulated with either EB or rMOMP. Mice immunized with PorB/VD1–2–4 produced higher IL-6 levels than any other of the Por/VD groups (Table 3). The highest IFN-γ and IL-6 levels were measured in T-cell supernatants from mice vaccinated with rMOMP, whereas those immunized with PBS did not secrete these cytokines.

**Table 3:** In vitro cytokine production by T cells from PorB/VD-immunized mice.

Immunization groups	EB stimulated		MOMP stimulated	
	IFN-γ (pg/ml) (mean ± 1 SE)	IL-6 (pg/ml) (mean ± 1 SE)	IFN-γ (pg/ml) (mean ± 1 SE)	IL-6 (pg/ml) (mean ± 1 SE)
PorB/VD1–3	349 ± 147^a^	12 ± 2^a^	193 ± 86^a,b^	19 ± 10^a,b^
PorB/VD1–4	125 ± 48^a,b^	<10 ± <10	21 ± 3^a,b^	11 ± 1^a,b^
PorB/VD1–3 + PorB/VD1–4	47 ± 15 ^a,b^	15 ± 8^a^	84 ± 26^a,b^	20 ± 8 ^a,b^
PorB/VD1–2–4	79 ± 24^a,b^	18 ± 4^a^	67 ± 20^a,b^	77 ± 13^a,b^
rMOMP/Z3–14	698 ± 225	25 ± 10	1601 ± 179	196 ± 45
PBS	<15 ± <15	<10 ± <10	<15 ± <15	<10 ± <10

^a^*P* < 0.05 by the Student’s *t*-test compared with the PBS group.

^b^*P* < 0.05 by the Student’s *t*-test compared with the rMOMP.

### Assessment of the protective effect of vaccination with PorB/VD1–3, PorB/VD1–4, and PorB/VD1–2–4 in a *C. muridarum* intranasal challenge mouse model

#### Body weight changes

**Fig. 2:**
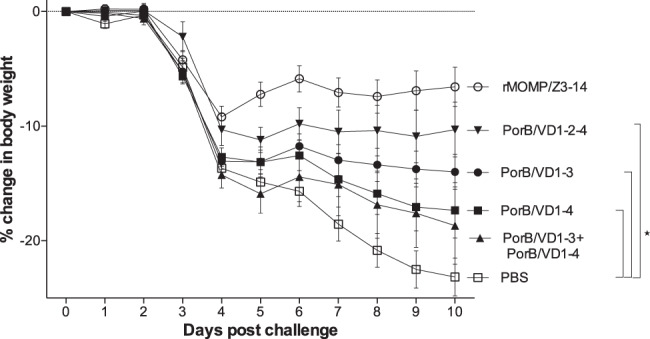
Daily percentage change in mean body weight following the i.n. challenge with *C. muridarum*. Daily percentage changes in mean body weight (±1 SE) following the i.n. challenge. Symbol *P < 0.05 by the repeated-measures ANOVA.

As a parameter of systemic disease, body weight changes following the i.n. challenge was monitored. Vaccinated and challenged mice were weighed daily until D10 p.c., when they were killed. As shown in Fig. 2, all mice, including the control, immunized mice (rMOMP and PBS) quickly lost weight from D2 p.c. through D4 p.c. Subsequently, the PBS-immunized mice continued to lose weight and those immunized with PorB/VD1–3 + PorB/VD1–4 and PorB/VD1–4 also lost substantial weight over time. Mice vaccinated with PorB/VD1–2–4 and PorB/VD1–3 maintained their body weight until D10 p.c. Mice immunized with rMOMP regained body weight starting at D5 p.c. Over the 10 d.p.c. period, changes in body weight between the PorB/VD1–3, PorB/VD1–4, and the PorB/VD1–2–4 groups versus the PBS control animals were significant by ANOVA (*P* < 0.05). In addition, differences in % body weight at D10 p.c. between the PorB/VD1–3 group (−14.00 ± 1.52), the PorB/VD1–2–4 group (−10.32 ± 2.42), vs. the PBS group (−23.17 ± 1.65) were also significant (*P* < 0.05) (Fig. 2 and Table 4). Mice immunized with rMOMP only lost −6.57 ± 1.77 of their initial body weight.

**Table 4:** Disease burden, yields of *C. muridarum* IFU, and IFN-γ and *C. muridarum*-specific IgA in lung’s supernatants at D10 p.c.

Vaccine	% Change body weight (mean ± 1 SE)	Lungs weight (g) (mean ± 1 SE)	Median number IFU recovered from lungs (min–max) × 10^6^	IFN-γ (pg/ml) (mean ± 1 SE)	IgA (OD_405_) (mean ± 1 SE)
PorB/VD1–3	−14.00 ± 1.52^a,b^	0.26 ± 0.01^a,b^	59 (4–672)^c,d^	2143 ± 186^b^	0.351 ± 0.009^a,b^
PorB/VD1–4	−17.35 ± 2.41^a,b^	0.28 ± 0.01^b^	66 (5–1374)^c,d^	2043 ± 235^b^	0.296 ± 0.013^a,b^
PorB/VD1–3 + PorB/VD1–4	−18.69 ± 3.38^b^	0.28 ± 0.01^b^	681 (5–9891)^d^	1762 ± 205^a,b^	0.338 ± 0.030^a,b^
PorB/VD1–2–4	−10.32 ± 2.42^a^	0.26 ± 0.01^a^	65 (0.3–1026)^c,d^	1528 ± 223^a^	0.480 ± 0.025^a,b^
rMOMP/Z3–14	−6.57 ± 1.77	0.22 ± 0.01	1 (0.02–228)	1051 ± 193	0.575 ± 0.030
PBS	−23.17 ± 1.65	0.29 ± 0.01	680 (158–9136)	2428 ± 117	0.201 ± 0.014

^a^*P* < 0.05 by the Student’s *t*-test compared with the PBS group.

^b^*P* < 0.05 by the Student’s *t*-test compared with the rMOMP group.

^c^*P* < 0.05 by the Mann–Whitney’s *U*-test compared with the PBS group.

^d^*P* < 0.05 by the Mann–Whitney’s *U*-test compared with the rMOMP group.

#### Lungs weight changes

**Fig. 3:**
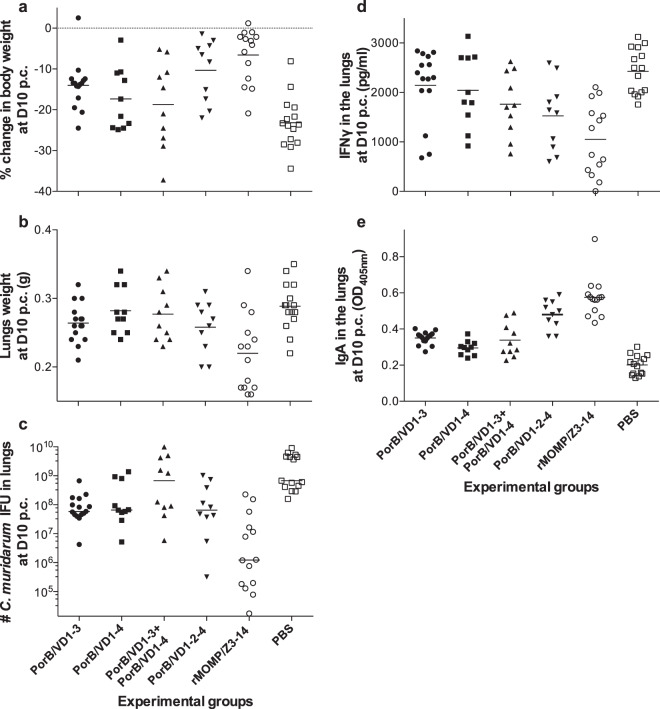
Local and systemic disease burden and local immune responses in lungs. **a** Percentage change in mean body weight following the i.n. challenge with *C. muridarum*. Change in body weight at D10 p.c. The mean is shown as a horizontal line. Each symbol represents a single animal. **b** Lungs weight at D10 p.c. with *C. muridarum*. Change in lungs weight at D10 p.c. The mean is shown as a horizontal line. Each symbol represents a single animal. **c** Number of IFUs recovered from lungs at D10 p.c. with *C. muridarum*. Number of *C. muridarum* IFU recovered from the lungs at D10 p.c. The median is shown as a horizontal line. Each symbol represents a single animal. **d** Amounts of IFN-γ in lung’s supernatants at D10 p.c. Levels of IFN-γ in lung’s supernatants at D10 p.c. The mean is shown as a horizontal line. Each symbol represents a single animal. **e** Amounts of *C. muridarum*-specific IgA in lung’s supernatants at D10 p.c. Levels of *C. muridarum*-specific IgA in lung’s supernatants at D10 p.c. The mean is shown as a horizontal line. Each symbol represents a single animal.

Lungs from vaccinated and challenged mice were collected at D10 p.c. and weighted as a parameter of local inflammatory responses (Fig. 3b and Table 4). Lungs from mice immunized with PorB/VD1–3 and PorB/VD1–2–4 were significantly lighter (mean weight in grams: 0.26 ± 0.01) than lungs from the PBS-negative control group (0.29 ± 0.01) (*P* < 0.05). The PorB/VD1–4 and PorB/VD1–3 + PorB/VD1–4 groups had the same lung weights (0.28 ± 0.01), which were not significantly different from that of the PBS control group. The weight of lungs from the rMOMP-positive control group was significantly lower than all the other groups (0.22 ± 0.01) (*P* < 0.05).

#### *C. muridarum* IFUs recovered from the lungs

The number of *C. muridarum* IFUs recovered from lungs collected at D10 p.c. was evaluated by culturing lung homogenates in HeLa cell monolayers (Fig. 3c and Table 4). The number of IFUs recovered from mice immunized with PorB/VD1–3 [59 (4–672) × 10^6^ IFUs] [median (range)], PorB/VD1–4 [66 (5–1374) × 10^6^], or PorB/VD1–2-4 [65 (0.3–1026) × 10^6^] were significantly lower than those from the PBS-negative control [680 (158–9136) × 10^6^] (*P* < 0.05), indicating protection. In contrast, from the PorB/VD1–3 + PorB/VD1–4 group, high number of IFUs were recovered [681 (5–9891) × 10^6^] (*P* > 0.05). rMOMP-vaccinated mice had very low number of IFUs [1 (0.02–228) × 10^6^].

### Levels of IFN-γ and *C. muridarum*-specific IgA in lungs supernatants

To correlate protection with local immune responses, supernatants from the lungs were collected to determine the levels of IFN-γ and *C. muridarum*-specific IgA (Table 4 and Fig. 3d, e). Levels of IFN-γ (pg/ml) in supernatants from mice immunized with the chimeric constructs PorB/VD1–2–4 (1528 ± 223) or PorB/VD1–3 + PorB/VD1–4 (1762 ± 205) were significantly lower than those from negative controls inoculated with PBS (2428 ± 117) (*P* < 0.05). Levels of IFN-γ from mice immunized with PorB/VD1–3 (2143 ± 186) or Por/VD1–4 (2043 ± 235) were not different from the PBS control (*P* > 0.05). In mice immunized with rMOMP, the level of IFN-γ was lower than in any other group (1051 ± 193) (*P* < 0.05).

Levels of *C. muridarum*-specific IgA (OD_405_) were significantly higher in lungs supernatants from mice vaccinated with the four chimeric constructs than those of mice immunized with PBS (0.201 ± 0.014) (*P* < 0.05). The highest level was found in mice vaccinated with PorB/VD1–2–4 (0.480 ± 0.025). IgA levels in supernatants from mice vaccinated with rMOMP (0.575 ± 0.030) were significantly higher than all other groups (*P* < 0.05).

## Discussion

The number of chlamydial infections continuous to increase throughout the world and there is an urgent need to implement a vaccine^1,2^. In this study, chimeric recombinant proteins, containing *C. muridarum* MOMP VDs, replacing the surface-exposed loops of the *N. lactamica* PorB, were used to immunize mice by mucosal and systemic routes using CpG-1826 and Montanide ISA 720 as adjuvants. Some of the constructs elicited significant humoral and cellular immune responses to *C. muridarum* EB and rMOMP. Mice vaccinated with these constructs were challenged in the nostrils and based on changes in body weight, weight of the lungs and number of *C. muridarum* IFU recovered from the lungs, mice were protected. To our knowledge, this is the first time that a chimeric vaccine using *N. lactamica* PorB as a scaffold to express *C. muridarum* MOMP VDs has been shown to elicit protection.

Several proteins including MOMP, CPAF, Pmps, and Pgp3, have been tested as vaccine antigens to protect mice against genital and respiratory challenges with *C. muridarum*^17,23,24,42^. In the genital model some of these antigens decreased vaginal shedding and inflammatory responses in the upper genital tract^44–46^. However, only rMOMP and nMOMP have elicited protection against long-term sequelae specifically infertility^40,47–49^. nMOMP can be produced in small quantities but escalating production to vaccinate humans will be technical difficult and costly. rMOMP, although not as efficacious at eliciting protection as nMOMP, can easily be produced in *E. coli*^41^. However, since MOMP is an intrinsic membrane protein it requires detergents or amphipols to keep it soluble in an aqueous solution^43,47^. The presence of eight Cys in MOMP also creates challenges to obtain an antigen that has consistent structural and antigenic properties.

To address these shortcomings, Olsen et al.^72,73^ constructed CTH522, a Cys-free chimeric recombinant *C. trachomatis* MOMP antigen. CTH522 consists of two components. The N-terminal includes most of *C. trachomatis* serovar D MOMP. The C-terminal are extended VD4 segments from serovars D, E, F, and G. The VD4 region of MOMP is rich in neutralizing epitopes and contains the highly conserved species-specific linear epitope LNPTIAG^52^. CTH522, adjuvanted with CAFO1, delivered by mucosal and systemic routes, elicits robust humoral and cell-mediated immune responses in mice and protects against shedding and inflammatory responses in the upper genital tract^72,73^. This chimeric recombinant MOMP has now successfully completed Phase 1 clinical trials^74^.

Using a different approach to obtain recombinant MOMP-based antigens, our group has previously designed constructs composed of the *N. lactamica* PorB porin in which surface-exposed loops were substituted with surface-exposed loops of MOMP, namely those including the VD and part of the adjacent CD regions^55^. Analysis of immune responses in mice to constructs containing individual *C. muridarum* MOMP loops showed high levels of MOMP-specific antibodies that cross-reacted with both rMOMP and nMOMP, and were recognized by antibodies raised against both rMOMP and nMOMP^55^. To determine whether increasing the number of MOMP loops expressed simultaneously would enhance such responses, multiple loop constructs were examined (P. Massari, unpublished results). Interestingly, variability was observed in the MOMP-specific cross-reactive immunity elicited by the multiple constructs. For example, constructs containing two loops combinations that included loop 2 (VD1) showed higher cross-reactivity with both nMOMP and rMOMP than other two-loop combinations (i.e., PorB/VD2–3, etc.). The strategy for designing our recombinant PorB/VD antigens was based on a conservative replacement of the PorB surface-exposed loops with MOMP loops that took into consideration the potential effect of such replacements on a few structural premises, such as amino acid sequence similarity, general loop charge and availability of suitable “anchor” residues for loop swapping. However, as the secondary, tertiary, and quaternary structures of MOMP are unknown, we could not compare these with PorB and therefore, insertion sites and fine structure details could not be optimized a priori^37^. However, all the PorB/VD combinations were immunogenic and recognized by anti-MOMP antibodies, and induced cross-reactive responses to both nMOMP and rMOMP. Here, the individual and the multiple MOMP loop swap constructs were examined in a mouse model of *C. muridarum* respiratory infections to evaluate their protective potential as vaccine candidates.

Mice were vaccinated with each of the PorB/VD constructs using an adjuvant combination and routes of immunization previously found to be protective in the respiratory and genital challenge model using rMOMP or nMOMP as antigens^40,41,48,49^. Due to a large number of constructs, a preliminary in vivo screen was carried out with a small number of mice to eliminate antigens that failed to induce protection; e.g., none of the individual loop swap constructs (e.g., PorB/VD1) elicited protection^55^. Three multiple loop swap constructs were identified that led to lower body weight changes after the challenge, decreased lungs weight and lower number of *C. muridarum* IFUs recovered from the lungs than in non-immunized mice. Based on these criteria, a protective function was assigned to the PorB/VD1–3, PorB/VD1–4, and PorB/VD1–2–4 constructs and the experiment was repeated with these groups and the controls.

We also tested a combination of PorB/VD1–3 + PorB/VD1–4. Antigen combinations can result in synergistic, additive, neutral, or antagonistic effects. For example, Finco et al.^75^ immunized mice with *C. muridarum* TC0106, TC0210, TC0313, or TC0741 and observed 0.5–0.9 log_10_ reduction in the number of IFUs recovered from the lungs. However, vaccination with a combination of the four antigens resulted in a 4.1 log_10_, decrease in the yield of *C. muridarum* from the lungs, indicative of additive effects. Others have reported neutral results when testing combinations of antigens in the respiratory model. For instance, Cheng et al.^66^ immunized mice with components of the *C. muridarum* putative ATP synthase complex TC0580, TC0581, TC0582, TC0584 singly or in combination with rMOMP. Animals immunized with combinations of these antigens were only protected as well as mice vaccinated with rMOMP, the most protective antigen in the formulation. Here, PorB/VD1–3 + PorB/VD1–4 did not elicite higher humoral immune responses that those induced by PorB/VD1–3 alone. Based on the results of the B-cell MOMP epitope mapping, immunizing with the two constructs suppressed the antibody responses to VD4. The inhibition of responses to VD4 may account for the decreased humoral responses and protection induced by this antigen combination. Other combinations may be explored in future studies, as well as placement of the MOMP loops into different PorB loops to enhance presentation of VD4.

A strong Th1-biased response has been observed when live EB are used as vaccine antigens. When testing subunit vaccines, such as MOMP, adjuvants significantly affect the results^40,70^. Th1 immune responses are expected when utilizing the CpG-1826 plus Montanide ISA 720 adjuvant combination^40,41^. Here, only the PorB/VD1–2–4 construct elicited Th1-biased responses when immune T cells were stimulated with either EB or rMOMP, while the other PorB/VD constructs induced Th2 responses. We can speculate that such discrepancies may be due to an intrinsic effect of each different constructs. It is important however to point out that PorB/VD1–2–4 induced the highest levels of neutralizing antibodies and the best protection.

Protective responses against *Chlamydia* infection are known to require a T-cell-mediated component^43,50^. To evaluate such responses in the mice vaccinated with the three constructs, the levels of IFN-γ and IL-6 in supernatants from T cells stimulated with EB or rMOMP were monitored. The highest levels of IFN-γ were elicited by PorB/VD1–3 in response to re-stimulation with EB or rMOMP. PorB/VD1–2–4 had the highest levels of IL-6 when stimulated with EB or rMOMP. This construct also had the highest levels of *C. muridarum*-specific IgA in lung supernatants and the lowest levels of IFN-γ. Thus, the characterization of the immune responses to PorB/VD1–3, PorB/VD1–4, and PorB/VD1–2–4, for the most part, paralleled the evidence of protection experiments. Indeed, the body weight, lungs weight and *C. muridarum* IFU recovered from lungs of vaccinated and challenged mice confirmed a protective effect of these recombinant antigens.

In conclusion, our results indicate that vaccination strategies against *Chlamydia* using our novel recombinant MOMP-based antigens may lead to an improvement of the current use of rMOMP. We realize however, that despite the protection elicited by our constructs it is not yet as robust as that induced by rMOMP. This approach, on the other hand, may present significant advantages over rMOMP for producing conformationally stable and immunogenic antigens^76^. In addition, it provides the opportunity to produce optimized antigens by for example, positioning the MOMP loops for an improved exposure of the VDs more closely mimicking the structural conformation of nMOMP. Furthermore, due to the trimeric nature of PorB and the interchangeable positioning of the MOMP loops within this protein, it will be possible to design constructs containing a given loop (X) multiple times [a PorB/VD(X) repeat]. Introducing T-cell epitopes from the CD of MOMP should enhance T-cell responses. Ultimately, to apply this strategy to a *C. trachomatis* vaccine, our optimized constructs will be suitable for expression of the same loop (Y) from different *C. trachomatis* serovars (up to four at the same time, [PorB/VD(Y)s repeat]) or any combination of MOMP loops X-Y-Z-W from different *C. trachomatis* serovars simultaneously, bypassing the shortcomings of using a mono-serovar antigen. We are in the process of designing such constructs. Potentially, this approach could also be used as a scaffold to deliver antigens from several sexually transmitted pathogens.

### Reporting summary

Further information on experimental design is available in the Nature Research Reporting Summary linked to this article.

## Supplementary information

Supplementary Information

Reporting Summary

## Data Availability

The authors confirm that all relevant data are included in the paper and its Supplementary Information.
