# Supplementary material for: Protection against a chlamydial respiratory challenge by a chimeric vaccine formulated with the Chlamydia muridarum major outer membrane protein variable domains using the Neisseria lactamica porin B as a scaffold
Source: NPJ Vaccines. 2020 May 8;5:37. doi: 10.1038/s41541-020-0182-9 (PMC7210953; doi:10.1038/s41541-020-0182-9)
Supplement: Supplementary file 1 — Supplementary Information [file 41541_2020_182_MOESM1_ESM.pdf]

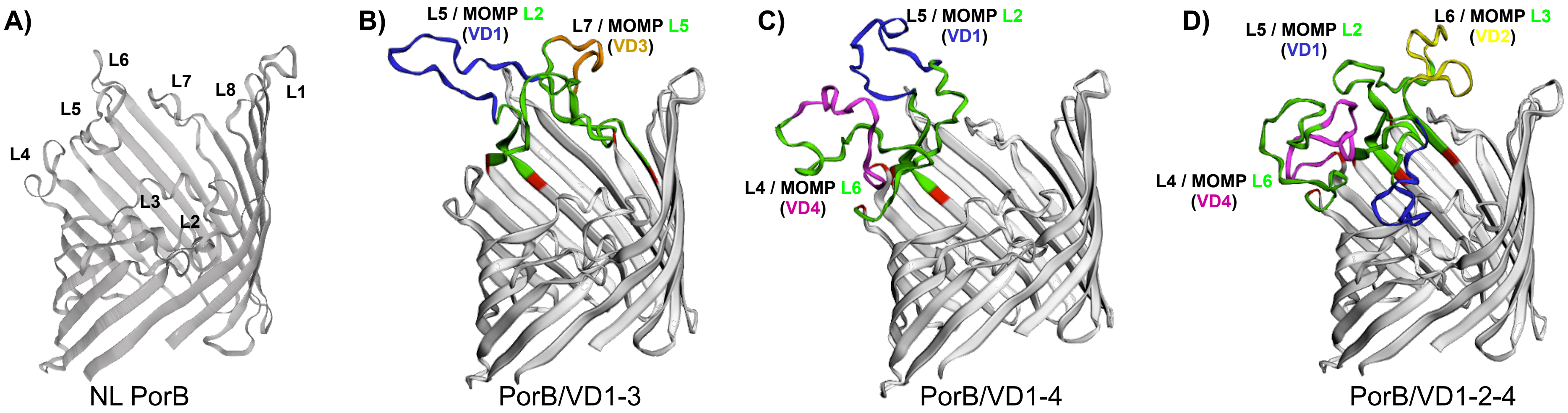

Supplementary Figure 1. Cartoon of the PorB monomer (front view) created with information from (62-66); **A)** Nlac PorB; loops 1 through 8 are indicated. **B)** PorB/VD1-3: loop 5 of PorB was replaced with MOMP loop 2 (green) containing the VD1 region (blue) and loop 7 of PorB was replaced with MOMP loop 2 (green) containing VD1 (orange). Anchor residues for loop exchange are depicted in red. **C)** PorB/VD1-4: loop 5 of PorB was replaced with MOMP loop 2 (green) containing the VD1 region (blue) and loop 4 of PorB was replaced with MOMP loop 6 (green) containing VD4 (purple). D) PorB/VD1-2-4: loop 5 of PorB was replaced with MOMP loop 2 (green) containing the VD1 region (blue), loop 6 of PorB was replaced with MOMP loop 3 (green) containing VD2 (yellow) and loop 6 of PorB was replaced with MOMP loop 6 (green) containing VD4 (purple).
